# PD22 - Examination about eosinophilia, cytokine and gastrointestinal allergy in the preterm infant

**DOI:** 10.1186/2045-7022-4-S1-P22

**Published:** 2014-02-28

**Authors:** Ogawa Tomomi, Fukuda Noriko, Junko Sawaki, Maiko Misaki, Minagawa Kyoko

**Affiliations:** 1 Hyogo Medical College, Hyogo, Japan

## Purpose

Several studies have shown the relationship between eosinophilia and gastrointestinal allergy in preterm infants; however, its mechanism has not yet been established. Interleukin (IL)-5 was shown to be involved in allergic inflammation by promoting eosinophil differentiation and activation and inducing basophil production. In this study, we examined the changes in eosinophil and basophil counts and the association with cytokines in low birth-weight infants showing a predominant symptom of abdominal distension with no serious symptoms such as blood in the stool and anaphylaxis. These infants were admitted to the neonatal intensive care unit (NICU) in our hospital for 1 week or more after birth.

## Methods

Blood eosinophil and basophil counts and serum IL-5, IL-4, and IL-10 levels were measured over time and compared with the initial levels in 17 low birth-weight infants showing abdominal distension, who were admitted to the NICU in our hospital between January 2008 and the end of March 2010. The cases were examined retrospectively, including the clinical background and onset of allergy symptoms. The consent for participation in this study was obtained from each infant’s family.

## Results

The mean gestational age was 30 weeks and 2 days (range, 24 weeks and 3 days to 38 weeks and 2 days). The mean birth weight was 1352.9 g (range, 454–2358 g). The mean age by the time of enteral feeding was 5.9 days and that by symptom onset was 20.8 days (range, 8–51 days). Sixteen of the 17 infants (94%) had eosinophilia (eosinophil count > 700/μl). Four of the 17 infants (23.5%) showed abnormally high eosinophil count (>3000/μl). The mean age for initiation of the increased eosinophil count was 22.3 days (range, 6–39 days). The mean peak eosinophil count was 2486.4/μl (range, 754–7980/μl). The serum IL-5 level was measured in 107 specimens. The eosinophil count was high in 4 infants (>4 pg/ml) who received milk during treatment. The IL-10 level tended to increase at an early stage after birth. In all the infants, serum IL-4 was less than or equal to the detection value. Immunoglobulin E (IgE) was positive in 6 infants. The final IgE was negative in the infants who received intervention by using milk as treatment.

## Conclusion

Abnormal increase in eosinophil counts and elevated serum IL-5 levels were observed in infants with abdominal distension, which was likely to lead to delayed allergic reactions. Elevation of serum IL-5 level was similar to the change in the medical conditions, which may be effective for determining disease pathogenesis and evaluating the medical conditions.

